# Retrospectively ECG-gated helical vs. non-ECG-synchronized high-pitch CTA of the aortic root for TAVI planning

**DOI:** 10.1371/journal.pone.0232673

**Published:** 2020-05-12

**Authors:** Barbora Horehledova, Casper Mihl, Ellen Boswijk, Genevieve A. J. C. Crombag, Estelle C. Nijssen, Patty J. Nelemans, Leo F. Veenstra, Joachim E. Wildberger, Marco Das

**Affiliations:** 1 Department of Radiology and Nuclear Medicine, Maastricht University Medical Center, Maastricht, The Netherlands; 2 CARIM School for Cardiovascular Diseases, Maastricht University Medical Center, Maastricht, The Netherlands; 3 Department of Epidemiology, Maastricht University Medical Center, Maastricht, The Netherlands; 4 Department of Cardiology, Maastricht University Medical Center, Maastricht, The Netherlands; Medical University Innsbruck, AUSTRIA

## Abstract

**Background:**

Multidetector computed tomography (MDCT) plays a key role in patient assessment prior to transcatheter aortic valve implantation (TAVI). However, to date no consensus has been established on what is the optimal pre-procedural imaging protocol. Variability in pre-TAVI acquisition protocols may lead to discrepancies in aortic annulus measurements and may potentially influence prosthesis size selection.

**Purpose:**

The current study evaluates the magnitude of differences in aortic annulus measurements using max-systolic, end-diastolic, and non-ECG-synchronized imaging, as well as the impact of method on prosthesis size selection.

**Material and methods:**

Fifty consecutive TAVI-candidates, who underwent retrospectively-ECG-gated CT angiography (CTA) of the aortic root, directly followed by non-ECG-synchronized high-pitch CT of the entire aorta, were retrospectively included. Aortic root dimensions were assessed at each 10% increment of the R-R interval (0–100%) and on the non-ECG-synchronized scan. Dimensional changes within the cardiac cycle were evaluated using a 1-way repeated ANOVA. Agreement in measurements between max-systole, end-diastole and non-ECG-synchronized scans was assessed with Bland-Altman analysis.

**Results:**

Maximal dimensions of the aortic root structures and minimum annulus-coronary ostia distances were measured during systole. Max-systolic measurements were significantly and substantially larger than end-diastolic (p<0.001) and non-ECG-synchronized measurements (p<0.001). Due to these discrepancies, the three methods resulted in the same prosthesis size selection in only 48–62% of patients.

**Conclusions:**

The systematic differences between max-systolic, end-diastolic and non-ECG-synchronized measurements for relevant aortic annular dimensions are both statistically significant and clinically relevant. Imaging strategy impacts prosthesis size selection in nearly half the TAVI-candidates. End-diastolic and non-ECG-synchronized imaging does not provide optimal information for prosthesis size selection. Systolic image acquisition is necessary for assessment of maximal annular dimensions and minimum annulus-coronary ostia distances.

## Introduction

Multi-detector row computed tomographic (MDCT) assessment of the aortic root plays an important role in pre-procedural planning of transcatheter aortic valve implantation (TAVI)[1–3]. Aortic root dimensions need to be accurately assessed in order to evaluate patient suitability for the procedure[4, 5], as well as for optimal prosthesis type and size selection[3, 5]. Precise prosthesis sizing is crucial for patient outcome: prosthesis under-sizing may result in paravalvular leakage, device migration or embolization[3, 6, 7], whereas significant over-sizing may increase the risk of post-procedural complications such as conduction disorders or annular rupture[3, 6–8].

The 2012 Society of Cardiovascular Computed Tomography (SCCT) guidelines for TAVI planning have not been conducive in establishing a golden standard pre-procedural MDCT protocol [3]. The guidelines state that, if available, assessment of maximal annular dimensions in systole may be preferable. However, to allow motion-free imaging, both retrospective ECG-gating and prospective ECG-triggering are considered adequate. The only prerequisite is that motion-free images of the aortic root have to be acquired. This ambiguity has led to the introduction of a variety of pre-TAVI protocols, in which aortic root dimensions are assessed at different time points of the cardiac cycle.

Although it is generally accepted that the aortic root is a dynamic structure, which constantly changes shape and dimensions throughout the cardiac cycle [2, 5, 6], the clinical relevance of these changes in patients with aortic stenosis is still a topic of debate [2, 4, 5, 7, 9]. Some TAVI groups use retrospectively ECG-gated protocols with image reconstruction during either systole [8, 10], diastole [11, 12], both systole and diastole [7, 9], or throughout the entire cardiac cycle [2, 6]. Other groups use prospectively ECG-triggered protocols, typically acquiring images during diastole [4, 13], but acquisition at the 40% phase of the R-R interval in patients with low cardiac rhythm frequencies (<65bpm) has also been reported [4].

The ambiguity has not been solved in the 2019 version of the SCCT guidelines, where the recommended acquisition method is dependent on manufacturer and/or type of CT-scanner [14]. Moreover, the use of ultra-high-pitch imaging, which offers the possibility to capture the whole heart, the aorta and peripheral access route within a single acquisition, is not even mentioned. High-pitch acquisition images are acquired at only one, frequently unspecified, time point of the R-R interval. Despite this, reduced scan times, together with potential reductions in contrast media (CM) volumes [15] and acquired radiation dose [16], have led to widespread use of high-pitch imaging in TAVI planning [15–17].

The aim of the current study is to describe the systematic and random discrepancies in aortic root measurements which occur when using max-systolic, end-diastolic, and non-ECG-synchronized high-pitch imaging, and to evaluate the impact on prosthesis size selection.

## Materials and methods

### Patient population

Pre-procedural imaging of fifty consecutive TAVI-candidates with severe and symptomatic aortic valve stenosis, who underwent pre-TAVI MDCT assessment of the aortic root between 01/2011 and 08/2012, were retrospectively included and assessed for aortic root measurements in 2018. Patients with a history of valve replacement were excluded from the data analysis.

All procedures performed in studies involving human participants were in accordance with the ethical standards of the institutional and/or national research committee and with the 1964 Helsinki declaration and its later amendments or comparable ethical standards. The approval for this study was obtained from the Institutional Review Board and the local medical ethical research committee (METC). Due to the retrospective nature of this study a waiver of written informed consent was issued by the Institutional Review Board. The data were coded and analyzed anonymously. The local METC (METC—Maastricht University Medical Center) reference number is METC:2018–0351.

### MDCT acquisition and CM protocol

All patients underwent a uniform dedicated scan protocol on a 2^nd^ generation dual-source MDCT (SOMATOM Definition Flash, Siemens Healthlineers, Forchheim, Germany): a low-pitch retrospective ECG-gated helical scan of the aortic root, followed by a high-pitch non-ECG-synchronized computed tomographic angiography (CTA) of the whole aorta. 120ml pre-warmed, monomeric, non-ionic, low osmolar iodinated CM (Iopromide, Ultravist 300, Bayer, Berlin, Germany) was injected according to a previously published CM protocol[6]. The acquisition and CM parameters are summarized in **Table 1**.

**Table 1:** Scan and contrast media application parameters.

	Retrospective ECG-gated protocol	Non ECG-synchronized high pitch protocol
Scan direction	Cranio-caudal	Cranio-caudal
Tube voltage [kV]	100	100
Tube current [mAs]	320	150
Dose modulation	CARE Dose4D	CARE Dose4D
CTDIvol [mGy]	62	5
Rotation time [s]	0.28	0.28
Temporal resolution [ms]	75	75
Pitch	0.17	3
Slice collimation	2x2x64x0.6	2x2x64x0.6
Slice width [mm]	0.75/0.7	1.5/1
Reconstruction kernel	B26f	I30f
Contrast media	Iopromide 300 (Ultravist)	
Test bolus	20ml CM * 7.2ml/s followed by 15ml NaCl* 7.2ml/s	
Main bolus	75ml CM * 7.2ml/s (100%)	
	50ml CM/NaCl * 7.2ml/s (50/50%)	
	25ml NaCl* 7.2ml/s	
Iodine delivery rate [gI/s]	2.16	

CM = contrast medium; ECG = electrocardiogram; gI = grams of iodine; kV = kilovolt; mAs = milliamper-second; mGy = milligray; ml = milliliter; mm = millimeter; NaCl = saline; s = second

### Image reconstruction

Scans were reconstructed using dedicated post-processing software (SyngoVia^TM^, Siemens, Forchheim, Germany) with a raw-data based iterative reconstruction algorithm (SAFIRE, Siemens Healthcare). The retrospectively ECG-gated scans were reconstructed for each 10% increment of the R-R interval (0–100%) to a 0.75mm slice thickness and a 0.7mm increment with a medium smooth fine noise (B26f) convolution kernel. Non-ECG-synchronized scans were reconstructed to a 1.5mm slice thickness and 1.0mm increment with a medium smooth fine noise (I30f) convolution kernel (SAFIRE, strength 2).

### Objective and subjective CT image quality

Objective image quality (IQ) was quantified in terms of attenuation in Hounsfield Units (HU) ± standard deviations (SD), signal-to-noise ratio (SNR), and contrast-to-noise ratio (CNR)[18]. Circular regions of interest (ROI) were manually placed at the level of the sinotubular junction, and, for noise and signal estimation, in the left ventricular myocardium. Objective IQ was considered diagnostic at vascular attenuation values>200HU[19, 20] and CNR>3[20, 21].

Subjective IQ, in terms of presence or absence of cardiac motion artifacts, was qualitatively evaluated by an experienced cardiovascular radiologist [Author 2] using a 4-point Likert scale: grade-1: non-diagnostic: impaired IQ precluding appropriate evaluation of the aortic root due to severe motion artifacts; grade-2: diagnostic: reduced IQ due to motion artifacts, but sufficient for aortic root dimension assessment; grade-3: good: presence of motion artifacts, but ability to reliably assess annular dimensions fully preserved; grade-4: excellent: complete absence of motion artifacts[18].

### Aortic annulus measurements

Pre-TAVI measurements were assessed in a blinded manner on the retrospectively ECG-gated scans at all 10%-time intervals of the cardiac cycle (0–100%) and on the non-ECG-synchronized scan. The anatomical structures of the aortic root were assessed in accordance with SCCT expert consensus guidelines[14]. Effective diameters, derived from the perimeter (D_P_) and annular area (D_A_), were calculated with commonly used formulas[18].

### Theoretical prosthesis sizing

Theoretical prosthesis size selection was based on the annular measurements assessed in the 20% phase (max-systole) and 70% phase reconstruction (end-diastole) on the retrospectively ECG-gated scan[6, 10, 11], and from the non-ECG-synchronized scan. To estimate the theoretical prosthesis size, industry guidelines were used (**S1 Appendix**) for the balloon-expandable (ESV; Edwards Sapien 3, Edwards Lifesciences Corp, Irvine, CA, USA) and for the self-expandable trans-catheter aortic valve (MCV; CoreValve Evolut R, Medtronic, Minneapolis, MN, USA). The ESV-guideline uses the annular area and D_A_ for prosthesis sizing whilst the MCV-guideline uses the annular diameter and perimeter. MCV-guidelines define the perimeter as: annulus diameter x π. Prosthesis sizing was done for both the measured perimeter and for the calculated perimeter (calculated-perimeter).

### Statistics

Statistical analysis was conducted using Statistical Package for Social Sciences version 23.0 (SPSS Inc., IBM Corp., Armonk, NY, USA). Categorical variables are expressed as frequencies and percentages. Continuous variables are expressed using mean values ±SD. Dimensional changes within the cardiac cycle are evaluated with a 1-way repeated ANOVA and Fischer’s Least Significant Difference post-hoc test. Agreement between imaging strategies is evaluated using Bland-Altman analysis. This analysis enables evaluation of the magnitude of discrepancies in size measurements between max-systolic versus end-diastolic /non-ECG-synchronized measurements. A paired t-test is used to calculate the mean difference between methods and the SD of the difference. The mean difference can be interpreted as systematic measurement error and the SD of the difference can be used for calculation of the 95% limits of agreement (LOA). The 95%LOA gives a direct indication of the degree of random measurement error and allows clinicians to assess whether the limits are small enough to be confident that one measurement method can replace another method. A two-sided p-value <0.05 was considered statistically significant. Agreement between suggested prosthesis sizes is expressed as the percentage of patients in which the same valve size would be selected based on two or all three different imaging protocols.

## Results

### Baseline characteristics

Baseline characteristics are summarized in **Table 2**. The study population consisted of 26 female (52%; 82±5 years) and 24 male (48%; 81±5 years) patients with an average age of 81±5 years.

**Table 2:** Baseline characteristics of the patient population.

	Mean±SD	Range
Gender		---
Age (years) (n = 50)	81±5	67–88
• female (n = 26; 52%) • male (n = 24; 48%)	• 82±5 • 81±5	• 67–88 • 71–88
Height [cm]	166±10	144–198
Weight [kg]	74 ±11	52–98
BSA [m^2^]	1.8±0.2	1.53–2.33
Aortic stenosis characteristic derived from TTE:		
Heart rate [bpm]	75 ±18	50–128
Ejection Fraction [%]	54±12	24–73
AVA [cm^2^]	0.81±0.2	0.4–1.6
Maximum pressure gradient [mmHg]	71±25	25–138
Mean pressure gradient [mmHg]	44±16	12–88

(AVA = aortic valve area; cm = centimeter; m = meter; mmHg = millimeters of mercury; n = number; SD = standard deviation; TTE-transthoracic echocardiography; % = percent)

### Objective and subjective CT image quality

The mean attenuation value of the retrospectively ECG-gated scans was 506±106HU, with mean SNR 13±4 and mean CRN 13±4. Cardiac motion artifacts were completely absent (grade-4) in 30 scans (60%), and non-significant (grade-3) artifacts occurred in 20 scans (40%). The mean attenuation value of the non-ECG-synchronized scans was 314±79HU, with mean SNR 13±3 and mean CRN 9±3. Cardiac motion artifacts were completely absent (grade-4) in 12 scans (24%), grade-3 artifacts occurred in 28 scans (56%), and grade-2 artifacts in 10 scans (20%).

### Aortic annulus measurements

A complete overview of mean annular dimensions as measured on retrospectively ECG-gated scans (0–100%) and non-ECG-synchronized scans are listed in **S2 Appendix**. The distances between the aortic annulus and the left and right coronary ostia were the only dimensions that reached their maximum during diastole (50%/60% phase) and their minimum during systole (10% phase). All other aortic annulus and aortic root dimensions reached their maximum dimensions in systole (10–30% phase).

**Fig 1:**
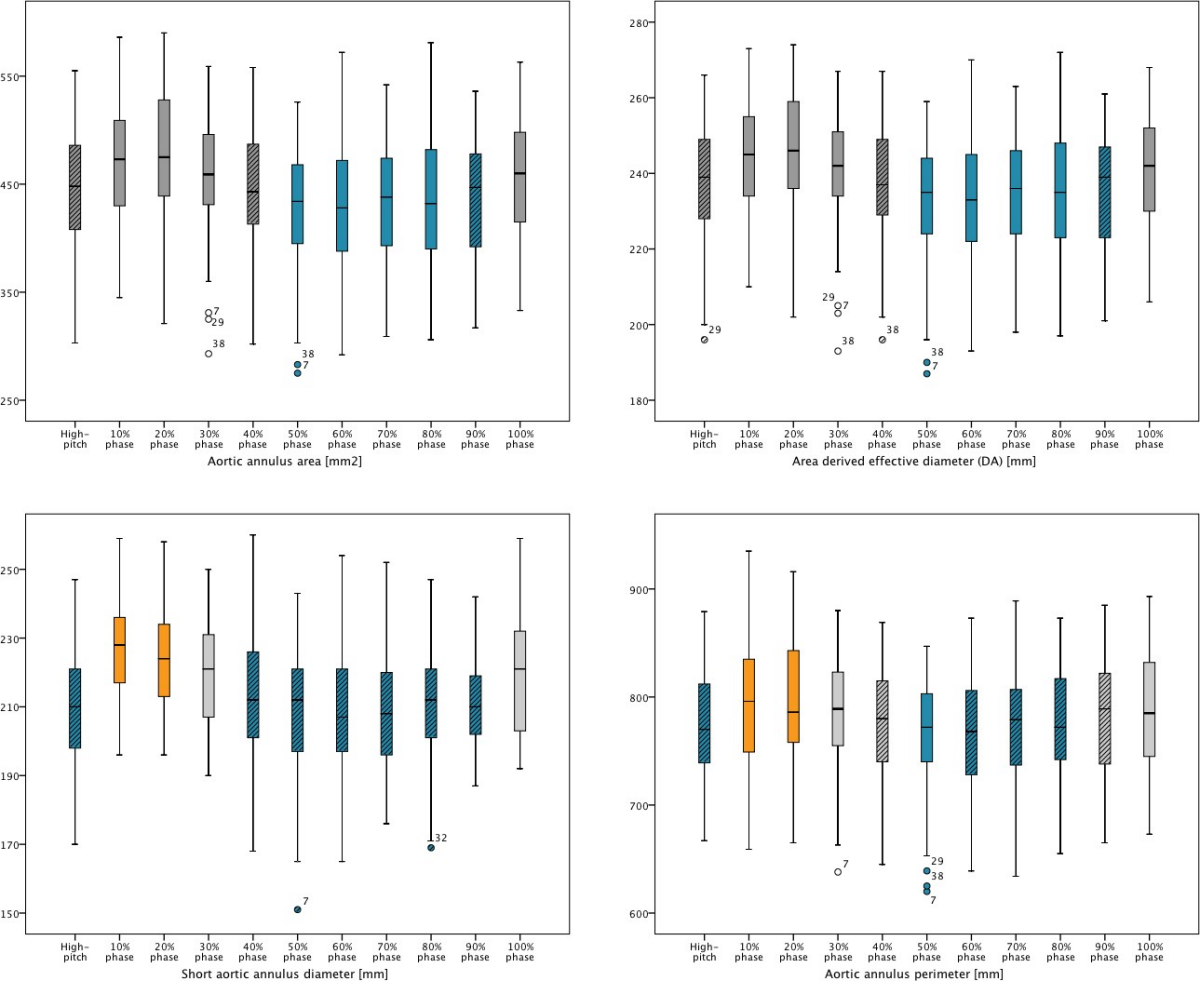
Box-plots showing dimensional changes in annular dimensions (10–100% phase) with correlation to non-ECG-synchronized high-pitch measurements. A) Area B) Area derived (effective) diameter (D_A_) C) Short diameter D) Measured perimeter. Orange box-plots indicate the max-systolic phases and blue box-plots the end-diastolic phases where dimensional changes are not statistically significant. Box-plots with a diagonal pattern indicate the phases in which measurements do not significantly differ to non-ECG-synchronized measurements.

**Fig 1** shows box-plots of dimensional changes in annular dimensions used for prosthesis size selection during the cardiac cycle. These demonstrate that the largest dimensions of the aortic annulus were measured during the max-systolic phases (10 and 20% phase), and that values decrease during diastolic phases. Max-systolic measurements were significantly larger than end-diastolic (p<0.001) and non-ECG-synchronized measurements (p<0.001).

Differences between two cardiac phases in max-systole (10 and 20%) for short diameter and measured perimeter were non-significant (p = 0.060 and p = 0.069, respectively; see orange box-plots in **Fig 1**). For annular area and D_A_ the maximum measurement in the 20% phase was significantly larger compared to measurements in all other phases.

Diastolic measurements did not significantly differ (p-values:0.065–0.736). The interval of non-significant dynamic differences in diastole (see blue box-plots in **Fig 1**) varied per annular dimension. There were no significant differences in measurements of annular area and D_A_ between 50–90% phases (p = 0.068–0.494), diastolic short diameter between 40–90% phases (p = 0.065–0.518), and measured perimeter between 50–80% phases (p = 0.071–0.736).

Non-ECG-synchronized values corresponded best with diastolic measurements. Annular area and D_A_ assessed on non-ECG-synchronized scans did not significantly differ from measurements at the 40% phase (p = 0.536 and p = 0.620, respectively; see diagonally structured box-plots in **Fig 1**) or 90% phase (p = 0.182 and p = 0.240, respectively). Non-ECG-synchronized short annular diameter did not significantly differ from measurements assessed in 40–90% phases (p = 0.311–0.977). Similarly, annular perimeter measured on non-ECG-synchronized scan did not significantly differ from that measured at 40% phase (p = 0.986) or 60–90% phases (p = 0.062–0.528).

The 20% and 70% phases were selected to represent max-systolic and end-diastolic measurements, respectively. **Table 3** shows results of the Blant-Altman analyses. A systematic difference between max-systolic, end-diastolic, and non-ECG-synchronized measurements of relevant annular dimensions. No proportional bias was found (p≥0.161). The 95% limits of agreement indicate the range wherein 95% of the discrepancies between two methods are situated. For example, for D_A_, max-systolic measurements may be 0.1mm below and up to 2.6mm above end-diastolic measurements, and for perimeter max-systolic measurements may be 1.7mm to 7.6mm above end-diastolic measurements.

**Table 3:** Aortic root dimensions used in prosthesis size selection.

Blant-Altman analysis shows the systematic and random discrepancies in aortic annulus measurements between max-systole minus A) end-diastole B) non-ECG-synchronized assessment				
**A)**	**Max-systole (20%) mean±SD**	**End-diastole (70%) mean±SD**	**Mean difference±SD**	**95% limits of agreement**
Short diameter (mm)	22.5±1.7	20.9 ±1.8	1.6±1.2	- 0.7–3.8
Area (mm^2^)	478.3±64	430.0±59	48.3±27.3	- 5.2–101.8
D_A_ (mm)	24.6±1.7	23.3±1.6	1.3±0.7	- 0.1–2.6
Perimeter (mm)	79.6±5.7	76.7±5.6	3.0±2.4	1.7–7.6
**B)**	**Max-systole (20%) mean±SD**	**Non-ECG-synchronized mean±SD**	**Mean difference±SD**	**95% limits of agreement**
Short diameter (mm)	22.5±1.7	20.9 ±1.7	1.5±1.4	-1.2–4.2
Area (mm^2^)	478.3±64	441.4±63	36.9±28.1	-18.1–91.9
D_A_ (mm)	24.6±1.7	23.6±1.7	1.0±0.7	-0.5–2.4
Perimeter (mm)	79.6±5.7	77.2±5.6	2.4±2.8	-3.0–7.8

(DA = area derived diameter; mm = millimeter; mm2 = square millimeter; SD = standard deviation)

The mean differences between measured perimeter and **calculated-perimeter** was 9.1mm (95%LOA: 2.2–16.0), 11.1mm (95%LOA: 2.8–19.5), and 11.5mm (95%LOA: 2.8–20.1) for max-systolic, end-diastolic and non-ECG-synchronized assessment respectively, with no proportional bias (p≥0.352).

### Theoretical aortic valve prosthesis sizing

Implications of observed measurement errors for theoretical prosthesis size selection were evaluated for both ESV (based on area and area-derived diameter) and MCV (based on diameter and perimeter). Pairwise comparison of max-systolic versus end-diastolic and non-ECG-synchronized measurements (**Table 4)** showed that, in case of discrepancies, end-diastolic and non-ECG-synchronized measurements always led to smaller prosthesis size selection. Pairwise comparison between end-diastolic and non-ECG-synchronized measurements (**S3 Appendix)** showed that the under-/over-sizing potential was not systematic between end-diastolic and non-ECG-synchronized measurements.

**Table 4:** Pairwise comparison of selected prosthesis sizes between the max-systolic versus end-diastolic and non-ECG-synchronized measurements.

• Green cells represent cases with agreement in prosthesis size between max-systole and end-diastole/non-ECG-synchronized measurement • Yellow cells represent cases where smaller prosthesis size would be selected based on end-diastolic/non-ECG-synchronized measurement compared to max-systolic measurement • Diagonally crossed cells indicate cases where unsuitable annular dimensions (too small/ too big) would be assessed									
**A) ESV-Annular Area**									
Max-systolic (20%)		End-diastolic (70%)				Non-ECG-synchronized			
	n.:	20mm	23mm	26mm	29mm	20mm	23mm	26mm	29mm
20mm	**1**	1	0	0	0	1	0	0	0
23mm	**11**	3	8	0	0	3	8	0	0
26mm	**30**	0	7	23	0	0	9	21	0
29mm	**8**	0	0	7	1	0	0	5	3
**B) ESV-D**_**A**_									
	n.:	20mm	23mm	26mm	29mm	20mm	23mm	26mm	29mm
20mm	**1**	1	0	0	0	1	0	0	0
23mm	**12**	3	9	0	0	2	10	0	0
26mm	**29**	0	6	23	0	0	6	23	0
29mm	**8**	0	0	7	1	0	0	5	3
**C) MCV-Short Annular Diameter**									
	n.:	23mm	26mm	29mm	Not-suitable	23mm	26mm	29mm	
23mm	**4**	4	0	0	1	3	0	0	
26mm	**31**	7	24	0	0	7	24	0	
29mm	**15**	0	8	7	0	0	9	6	
**D) MCV–Perimeter-Measured**									
	n.:	23mm	26mm	29mm	Not-suitable	23mm	26mm	29mm	Not-suitable
23mm	**0**	0	0	0	0	0	0	0	0
26mm	**5**	0	5	0	0	0	5	0	0
29mm	**27**	0	6	21	0	0	5	22	0
Not-suitable	**18**	0	0	9	9	0	0	6	12
**E) MCV–Calculated -perimeter**									
	n.:	23mm		26mm	29mm	Not-suitable	23mm	26mm	29mm
23mm	**5**	5		0	0	1	4	0	0
26mm	**31**	9		22	0	0	7	24	0
29mm	**14**	0		8	6	0	0	9	5

(DA = area derived diameter; ESV = Edwards Sapien Valve; MCV = Medtronic Core Valve)

When based on annular area, non-ECG-synchronized and end-diastolic measurements led to a selection of **ESV** one size smaller in 17 patients (34%) compared to max-systolic measurements. When based on D_A_ and using end-diastolic and non-ECG-synchronized measurements, one size smaller ESV would be selected in 16 (32%) and 13 (26%) patients respectively, compared to max-systolic measurements.

When based on short diameter, end-diastolic and non-ECG-synchronized measurements led to a selection of **MCV** one size smaller in 15 (30%) and 17 (34%) patients respectively, compared to max-systolic sizing. One patient would be considered unsuitable for a TAVI procedure based on the non-ECG-synchronized short annular diameter of 17.0mm, while max-systolic and end-diastolic measurements would lead to selection of 23mm MCV valve.

Max-systolic, end-diastolic and non-ECG-synchronized measurements would result in selection of the same **MCV** prosthesis size in 24 patients (48%) based on measured perimeter, and in 27 patients (54%) based on calculated-perimeter. Nine patients (18%) would be considered unsuitable for a TAVI using all three protocols, as the max-systolic, end-diastolic and non-ECG-synchronized perimeter measurements were all greater than 81.7mm. An additional 9 patients would be excluded from TAVI based on the max-systolic measured perimeter. Compared to the max-systolic assessment, one size smaller MCV would be selected for 6 patients (12%) based on the end-diastolic measurement, and for 5 (10%) patients based on the non-ECG-synchronized perimeter measurement.

## Discussion

Different cardiac imaging strategies result in discrepant aortic annulus measurements, affecting the theoretical selection of TAVI prosthesis size in almost half the patients, regardless of prosthesis type. Furthermore, the annular dimension chosen for the prosthesis size selection can additionally influence disagreement between acquisition methods or even limit suitability of the patient for a TAVI procedure. To the best of our knowledge, this is the first study to evaluate the differences between aortic annulus measurements derived from the retrospectively-ECG-gated and non-ECG-synchronized high-pitch MDCT scans in light of prosthesis size selection.

Our results contradict the study by Bertaso et.al., who found that differences in annular dimensions measured during systole and diastole were unlikely to alter patient suitability for TAVI or clinical decision making regarding prosthesis size[5]. In their study a disagreement in prosthesis size was observed in only 2 of 34 patients (6%) between diameters measured at systole and diastole for an older type of self-expandable valve. Remarkably, the discrepant systolic diameter resulted in a smaller prosthesis size compared to the diastolic diameter. Our findings concur with a large multicenter study by Murphy et.al., which led to the conclusion that even small differences in annular area and perimeter measured at systole and diastole can result in under-sizing. This occurred in 247 of 507 patients (49%) using the adapted industry sizing thresholds for an older generation balloon-expandable valve[9]. The systematic potential for under-sizing in diastole was confirmed in our study, for both MCV and the newer generation ESV.

Precise assessment of maximal aortic root dimensions and the minimum distance between the annulus and coronary ostia are desirable for TAVI planning in order to minimize the risk of post-procedural paravalvular leakage and/or coronary ostium occlusion [3]. Although the 2019 SCCT guidelines state provide no recommendation whether the coronary ostia height should be measured in systole or diastole [14], the results of the current study show that the largest aortic annulus dimension and also the smallest annulus-coronary ostia distances are ideally measured during max-systole.

Despite the fact that non-ECG-synchronized annular measurements relevant for prosthesis sizing were best correlated to the diastolic phases of the cardiac cycle, the prosthesis sizes derived from the non-ECG-synchronized measurements differed to those based on end-diastolic measurements in 18–32% of patients, depending on annular dimension or prosthesis type (**S3 Appendix**). This shows that even small dynamic changes in annular dimensions during diastole influence prosthesis sizing, despite often being statistically non-significant. Systematic discrepancies between max-systolic and end-diastolic measurements in the current study are described separately for each prosthesis sizing dimension. Estimating the dynamism of the aortic annulus dimension is helpful in cases where the assessment of maximal annular dimensions in systole is not possible or technically feasible.

Annular dimensions used for prosthesis sizing significantly affect the degree of prosthesis size agreement between max-systolic, end-diastolic and non-ECG-synchronized protocols. In this study, a higher agreement was observed in the theoretically selected prosthesis size when sizing was based on annular area or D_A_ (54% and 62% of cases, respectively). The annular perimeter has often been proposed as the best-suited annular dimension for prosthesis size selection, due to negligible dimensional changes in patients with aortic stenosis[2, 3]. However, in the MCV sizing guidelines only the calculated-perimeter is used. In the current study the measured perimeter was much larger than the calculated-perimeter (mean difference 9-12mm; p<0.001). Circularity is assumed for the calculation of the perimeter from the diameter (D_P_)[7]. However, aortic annulus is an elliptical structure, therefore, such calculations cannot be used to reliably describe annular dimensions.

The main advantage of prospective ECG-triggered (end-diastolic) and non-ECG-synchronized imaging is the shorter image acquisition time compared to retrospective ECG-gating, which potentially reduces both CM volume and radiation dose[15]. The first is of special interest, because TAVI-candidates often present with comorbidities including impaired renal function[15]. There is growing evidence, however, that the risk of acute kidney injury associated with intra-venous CM application is likely overestimated [22], even in patients with impaired kidney function[23]. Still, reasonable CM management should be pursued. Other MDCT strategies than non-ECG-synchronized acquisition are available, allowing decreased CM volumes. For example, significant CM volume reduction (34–67%) can be achieved in pre-TAVI CTA with the use of a low kV protocol (80 kVp)[20]. Since radiation induced malignancies usually develop 10–20 years after exposure [24], the reduction of acquired radiation dose is not a main focus in TAVI-candidates whose average age is 80 years[6, 8, 14, 15] with a mean survival of two to three years[25]. Nevertheless, radiation dose can be reduced through tube current modulation or lower tube potential protocols[26, 27], even on older MDCT scanners[28]. Although max-systolic retrospectively ECG-gated imaging might not result in the lowest possible radiation and CM dose, these drawbacks do not outweigh the benefits. The assessment of maximal annular dimensions during max-systole is crucial in order to address the risk of post-procedural complications associated with prosthesis under-estimation[3, 29]. Moreover, the results of this study also show that retrospectively-ECG-gated acquisition produces less cardiac motion artifacts in the aortic root region than non-ECG-synchronized imaging.

The TAVI-candidate population has a multitude of intrinsic characteristics such as multiple comorbidities and a variety in presence and degree of annular calcifications at the stenotic valve. These patient characteristics cannot be influenced. On the other hand, the current study shows that variability in pre-TAVI imaging and sizing dimensions can also significantly influence clinical decisions. These differences resulting from imaging protocols are a variable in TAVI planning which can and should be taken out of the equation through standardization.

### Limitations

This is a single-center retrospective study of a relatively small number of TAVI-candidates, which may not have been a representative sample of the general TAVI population. A gold standard reference protocol for pre-TAVI MDCT assessment is non-existent. Since we only evaluated the current industry recommendations for self- and balloon-expandable trans-catheter devices, our results may not be extrapolated to valves of other manufacturers or different type of deployment. For the purpose of this study, we assumed that the prosthesis would expand to its nominal size. The retrospective character of this study does not allow the comparison of clinical outcome or severity of paravalvular leakage between ECG-gated and high-pitch acquisition. Clinical outcome and sizing related complications were not investigated. This should be addressed in future studies.

## Conclusion

The systematic differences between max-systolic, end-diastolic and non-ECG-synchronized measurements for relevant aortic annulus dimensions are both statistically significant and clinically relevant. End-diastolic and non-ECG-synchronized high-pitch imaging does not provide optimal information for TAVI planning and prosthesis size selection. Systolic image acquisition is necessary in order to obtain maximal annular dimensions and smallest annulus-coronary ostia distances.

## Supporting information

S1 Appendix Manufacturers prosthesis sizing guidelines. (mm = millimeter; mm^2^ = square millimeter) * perimeter = diameter x π.
(PDF)

S2 Appendix Mean aortic annulus structures measured at each 10% increment of the R-R interval (10–100% phase) and on non-ECG-synchronized scans. (D_A_ = area derived diameter; D_P =_ perimeter derived diameter; LCA = distance from annulus to left coronary artery ostium; RCA = distance from annulus to left coronary artery ostium; STJ = sinotubular junction).
(PDF)

S3 Appendix Pairwise comparison of selected prosthesis sizes between the end-diastolic versus non-ECG-synchronized measurements. Green cells represent cases with agreement in prosthesis size between end-diastole and non-ECG-synchronized measurementYellow cells represent cases where smaller prosthesis size would be selected based on non-ECG-synchronized measurement compared to end-diastolic measurementBlue cells represent cases where larger prosthesis size would be selected based on non-ECG-synchronized measurement compared to end-diastolic measurementDiagonally crossed cells indicate cases where unsuitable annular dimensions (too small/ too big) would be assessed.
(PDF)

S1 File (DOCX)
