# Supplementary material for: Retrospectively ECG-gated helical vs. non-ECG-synchronized high-pitch CTA of the aortic root for TAVI planning
Source: PLoS One. 2020 May 12;15(5):e0232673. doi: 10.1371/journal.pone.0232673 (PMC7217477; doi:10.1371/journal.pone.0232673)
Supplement: S1 Appendix — (mm = millimeter; mm2 = square millimeter) * perimeter = diameter x π. (PDF) [file pone.0232673.s001.pdf]

S1 Appendix Table 1: Manufacturers prosthesis sizing guidelines

Balloon expandable valve (Edwards Sapien 3)

Valve Size (= Device Diameter)	**20mm**	**23mm**	**26mm**	**29mm**
	Suitable Annular Dimensions			
Aortic Annulus Area [mm^2]	273-345	338-430	430-546	540-683
Effective diameter (area) (D_A) [mm]	18.6-21	20.7-23.4	23.4.-26.4	26.2-29.5

Self-expandable valve (Medtronic CoreValve Evolut R)

Valve Size (= Device Diameter)	**23mm**	**26mm**	**29mm**
	Suitable Annular Dimensions		
Aortic Annulus Diameter [mm]	18-20	20-23	23-26
Aortic Annulus Perimeter* [mm]	56.5-62.8	62.8 -72.3	72.3-81.7

(mm = millimeter; mm^2 = square millimeter)

* perimeter = diameter x π
