# Supplementary material for: Retrospectively ECG-gated helical vs. non-ECG-synchronized high-pitch CTA of the aortic root for TAVI planning
Source: PLoS One. 2020 May 12;15(5):e0232673. doi: 10.1371/journal.pone.0232673 (PMC7217477; doi:10.1371/journal.pone.0232673)
Supplement: S2 Appendix — (DA = area derived diameter; DP = perimeter derived diameter; LCA = distance from annulus to left coronary artery ostium; RCA = distance from annulus to left coronary artery ostium; STJ = sinotubular junction). (PDF) [file pone.0232673.s002.pdf]

S2 Appendix Table 2: Mean aortic annulus structures measured at each 10% increment of the R-R interval (10-100% phase) and on non-ECG-synchronized scans

	10% phase	20% phase	30% phase	40% phase	50% phase	60% phase	70% phase	80% phase	90% phase	100% phase	Non-ECG-synchronized
Short diameter	22.73±1.71	22.46±1.68	21.99±1.71	21.17±1.98	20.96±1.92	20.92±1.73	20.87±1.8	20.92±1.86	21.05±1.43	22.00±1.95	20.93±1.73
Long diameter	27.61±2.13	27.85±2.05	27.81±2.04	27.66±2.13	27.66±2.3	27.70±2.21	27.44±2.22	27.65±2.27	27.77±2.23	27.67±2.36	27.76±2.17
Perimeter	79.03±5.72	79.64±5.74	78.44±5.6	77.31±5.5	76.31±5.72	76.53±5.59	76.68±5.63	77.04±5.37	77.82±5.56	78.41±5.63	77.22±5.59
D_P	25.15±1.82	25.35±1.83	24.97±1.78	24.61±1.75	24.29±1.83	24.36±1.78	24.41±1.8	24.52±1.71	24.77±1.77	24.96±1.79	24.58±1.79
Area	467.28±60.66	478.32±63.92	455.06±61.17	439.62±58.57	426.65±60.74	427.48±60.18	430.0±58.55	431.98±62.2	435.26±58.31	455.66±59.3	441.4±62.74
D_A	24.34±1.60	24.62±1.66	24.01±1.67	23.61±1.61	23.24±1.72	23.27±1.66	23.34±1.63	23.39±1.70	23.49±1.60	24.04±1.59	23.64±1.72
RCA	17.40±2.71	17.82±2.67	17.89±2.60	18.05±2.90	18.33±3.48	18.06±3.02	18.22±2.92	18.22±2.65	18.27±2.67	18.07±2.90	17.74±2.47
LCA	16.43±2.34	16.44±2.47	16.63±2.50	16.75±2.74	16.82±2.50	17.22±2.28	16.74±2.21	17.04±2.31	16.68±2.52	16.48±2.15	16.03±2.20
STJ diameter	27.62±2.54	27.97±2.52	27.89±2.30	27.8±2.48	27.94±2.49	27.75±2.28	27.76±2.32	27.72±2.41	27.55±2.33	27.64±2.34	28.1±2.17
Widest portion of aortic root	34.9±3.49	35.25±3.47	35.2±3.43	35.13±3.28	34.8±3.35	34.7±3.45	34.54±3.29	34.32±3.29	34.53±3.48	34.47±3.45	33.79±3.13
Right ostium diameter	30.83±3.37	30.79±3.44	31.19±3.19	30.96±3.16	30.67±3.03	30.52±3.20	30.31±3.20	30.43±3.13	30.44±3.21	30.64±3.44	30.65±3.53
Left ostium diameter	32.14±3.21	33.32±3.27	33.13±3.47	33.08±3.17	32.46±3.05	32.54±3.37	32.45±3.2	32.55±3.32	32.7±3.32	32.17±3.52	33.08±3.23
Left cusp lenght	15.79±1.87	16.10±1.75	16.13±1.66	16.09±1.62	15.95±1.83	16.23±1.82	16.0±1.80	16.14±1.84	15.97±1.99	15.97±2.12	15.83±1.77
Right cusp lenght	15.86±1.63	16.26±1.78	15.66±1.54	15.62±2.01	15.46±1.80	15.75±1.67	15.62±1.78	15.5±1.54	15.69±1.69	16.07±1.55	15.71±1.81

(D_A = area derived diameter ; D_P=perimeter derived diameter ; LCA = distance from annulus to left coronary artery ostium ; RCA = distance from annulus to left coronary artery ostium; STJ = sinotubular junction)
