# Supplementary material for: Retrospectively ECG-gated helical vs. non-ECG-synchronized high-pitch CTA of the aortic root for TAVI planning
Source: PLoS One. 2020 May 12;15(5):e0232673. doi: 10.1371/journal.pone.0232673 (PMC7217477; doi:10.1371/journal.pone.0232673)
Supplement: S3 Appendix — Green cells represent cases with agreement in prosthesis size between end-diastole and non-ECG-synchronized measurementYellow cells represent cases where smaller prosthesis size would be selected based on non-ECG-synchronized measurement compared to end-diastolic measurementBlue cells represent cases where larger prosthesis size would be selected based on non-ECG-synchronized measurement compared to end-diastolic measurementDiagonally crossed cells indicate cases where unsuitable annular dimensions (too small/ too big) would be assessed. (PDF) [file pone.0232673.s003.pdf]

S3 Appendix Table 3: Pairwise comparison of selected prosthesis sizes between the end-diastolic versus non-ECG-synchronized measurements

- Green cells represent cases with agreement in prosthesis size between end-diastole and non-ECG-synchronized measurement
- Yellow cells represent cases where smaller prosthesis size would be selected based on non-ECG-synchronized measurement compared to end-diastolic measurement
- Blue cells represent cases where larger prosthesis size would be selected based on non-ECG-synchronized measurement compared to end-diastolic measurement
- Diagonally crossed cells indicate cases where unsuitable annular dimensions (too small/ too big) would be assessed

A) ESV-Annular Area

End-diastolic (70%)		Non-ECG-synchronized			
	n.:	20mm	23mm	26mm	29mm
20mm	**4**	3x	1	0	0
23mm	**15**	1	11	3	0
26mm	**30**	0	5	23	2
29mm	**1**	0	0	0	1

B) ESV- Area Derived Diameter (D_A)

End-diastolic (70%)		Non-ECG-synchronized			
	n.:	20mm	23mm	26mm	29mm
20mm	**4**	3	1	0	0
23mm	**15**	0	12	3	0
26mm	**30**	0	3	25	2
29mm	**1**	0	0	0	1

C) MCV-Short Annular Diameter

End-diastolic (70%)		Non-ECG-synchronized			
	n.:	Not-suitable	23mm	26mm	29mm
23mm	**11**	1	7	3	0
26mm	**32**	0	3	25	4
29mm	**7**	0	0	5	2

D) MCV– Perimeter-Measured

End-diastolic (70%) phase		Non-ECG-synchronized phase			
	n.:	23mm	26mm	29mm	Not-suitable
23mm	**0**	0	0	0	0
26mm	**11**	0	8	3	0
29mm	**30**	0	2	25	3
Not-suitable	**9**	0	0	0	9

E) MCV– Perimeter-Calculated

End-diastolic (70%)		Non-ECG-synchronized			
	n.:	Not-suitable	23mm	26mm	29mm
23mm	**14**	1	9	4	0
26mm	**30**	0	2	26	2
29mm	**6**	0	0	3	3
