# Supplementary material for: Retrospectively ECG-gated helical vs. non-ECG-synchronized high-pitch CTA of the aortic root for TAVI planning
Source: PLoS One. 2020 May 12;15(5):e0232673. doi: 10.1371/journal.pone.0232673 (PMC7217477; doi:10.1371/journal.pone.0232673)
Supplement: S1 File — (DOCX) [file pone.0232673.s004.docx]

| **S1 File: Abbreviations:** |
| --- |
| CM contrast media  CNR contrast-to-noise ratio CTA computed tomographic angiography D_A_ effective diameter derived from aortic annulus area D_P_ effective diameter derived from aortic annulus perimeter  ESV Edwards Sapien Valve HU Hounsfield Units  IQ image quality LOA limits of agreement  METC medical ethical research committee MCV Medtronic Core Valve  MDCT Multi-detector row computed tomography  ROI region(s) of interest SCCT Society of Cardiovascular Computed Tomography  SNR signal-to-noise ratio  TAVI Transcatheter aortic valve implantation |
